# Does China’s centralized volume-based drug procurement policy facilitate the transition from imitation to innovation for listed pharmaceutical companies? Empirical tests based on double difference model

**DOI:** 10.3389/fphar.2023.1192423

**Published:** 2023-05-30

**Authors:** Yang Gu, Qian Zhuang

**Affiliations:** School of International Pharmaceutical Business, China Pharmaceutical University, Nanjing, China

**Keywords:** China’s centralized volume-based procurement policy, imitation to innovation transition, double difference, innovation input, innovation output

## Abstract

**Introduction:** The normalized implementation of the centralized volume-based procurement policy for pharmaceuticals is a concerted push for supply-side structural reform of the pharmaceutical industry in China. The impact of the centralized drug procurement policy on pharmaceutical companies' transition from imitation to innovation is investigated to test whether a positive effect occurs in the innovation landscape of the pharmaceutical market.

**Methods:** The double difference method and a series of robustness tests were used based on data from a sample of listed pharmaceutical companies in Shanghai and Shenzhen A-shares between 2015 and 2021.

**Results:** The study found that the centralized drug procurement policy significantly contributed to the increased intensity of innovation input in the Chinese pharmaceutical industry. In terms of regional and firm nature heterogeneity, it was found that firms in the seven provinces belonging to the three economic regions had a better increase in innovation input intensity than other regions. Firms of state-owned nature had a better increase in innovation input intensity than private companies. The mechanism test found a partial mediating effect of nearly 10% for the cost of sales rate on the innovation input intensity of listed companies and a negative mediating effect on corporate operating profit.

**Discussion:** Further research found that the effect of centralized drug procurement policy on the improvement of innovation quality of listed pharmaceutical companies was evident. The innovation development of Chinese pharmaceutical companies no longer focused on the accumulation of innovation quantity.

## 1 Introduction

The centralized drug procurement system results from a game and compromises between many stakeholders. It has effectively reduced the burden of drugs on the public and the cost of drug distribution since its introduction. Internationally, the Group Purchasing Organizations (GPOs) in the United States have attracted many medical institutions to join the system, enhancing negotiating power of GPOs, giving it the upper hand in price negotiations with pharmaceutical suppliers (Burns, 2022). In New Zealand, multiple suppliers are invited to increase competition between themselves on the price of certain drugs, causing suppliers to voluntarily lower their prices. Australia’s direct government procurement model also has some positive signs in controlling drug prices and distribution links (Vitry, 2014). In India, a centralized bidding and purchasing agency under the government is responsible for procuring and distributing medicines from the essential medicines list, reducing the number of drug distribution links, and lowering suppliers’ drug distribution costs (Kaur et al., 2021). In China, the drug procurement system has roughly gone through three stages: independent procurement by hospitals, bidding and procurement by some provinces, and alliance and national centralized Volume-Based Procurement (the three stages are not completely fixed and clear, but may have some nuances or variations.), forming a working mechanism with a government organization, alliance procurement, and platform operation as the core. It plays an essential role in many aspects. For example, it was guiding drug prices back to a reasonable level, alleviating the burden of drug use on the public, bringing into play the strategic purchase of health insurance funds, promoting the reform of public healthcare institutions, and collaborating to promote the supply-side reform of China’s pharmaceutical industry. In 2018, the China Medical Security Bureau was set up to coordinate the implementation of the policy of state-organized centralized Volume-Based drug procurement and has carried out successively seven drug procurement exercises between 2018 and 2022. The prices of the selected drugs have dropped significantly, saving over RMB 50 billion in pharmaceutical costs each year. The experience of several rounds of centralized Volume-Based procurement shows that China’s centralized drug procurement policy has achieved significant price reduction and cost control. It promoted the reform objective of “exchanging quantity for price and squeezing out inflated price” in drug procurement.

However, it is not enough to consider only drug price and distribution costs in promoting centralized drug procurement. There is a potential risk of causing chaos in the pharmaceutical market, affecting business operations, and breaking the industry’s order of competition and development. China’s supply-side structural reform and the centralized drug procurement policy are being promoted in tandem. So it is essential to regulate the order of competition in centralized drug procurement, protect the interests of enterprises, maintain the healthy development of the industry, and achieve a win-win situation for multiple stakeholders. Pharmaceutical companies, as the source of vitality in the pharmaceutical market, are the source of the supply chain of centralized Volume-Based procurement, are significant stakeholders in the supply-side reform of the pharmaceutical market guided by the centralized Volume-Based procurement policy. The study of pharmaceutical companies’ performance, innovative vitality, and development prospects are of great significance to the future evolution of China’s centralized Volume-Based procurement system.

This study has important practical and theoretical significance: in terms of practical significance, the starting point of the centralized drug procurement policy was to encourage innovation among enterprises, prompting pharmaceutical companies to realize that innovation is the lifeline and to move from generic drugs to innovative drugs, improved new drugs, and high-quality generics. However, it is also a reality that the share prices of the pharmaceutical sector plummeted during the implementation of the policy, with several pharmaceutical companies losing market share overnight and causing dramatic upheaval in the industry. So, is the centralized Volume-Based drug procurement promoting the transformation of generic innovation in pharmaceutical companies, and is the industry turmoil a “short pain” or a “long pain”? Is there an increase in investment in innovation to achieve generic transformation? What are the mechanisms of impact? Is there heterogeneity of property rights among the selected pharmaceutical companies? Is there regional heterogeneity in the innovation transformation of selected pharmaceutical enterprises? Suppose there is an increase in innovation investment. What is the performance of the innovation strategies and output results of the selected pharmaceutical enterprises, and is there an emphasis on “quality” or “quantity,” or both “quality and quantity”? The study of these questions will integrate more stakeholder needs for the institutional design of the collection policy and provide experience for pharmaceutical companies to choose their innovation strategies. From a theoretical perspective, the existing literature has mainly explored the impact of centralized Volume-Based procurement policies on the risk of drug shortages, supply stability, the actual operation of the platform, hospitals, and patients, as well as the impact on drug prices and procurement volumes, and the selection of varieties. This paper empirically investigates the relationship between the centralized volume-based drug procurement policy and the innovation of pharmaceutical companies from the perspectives of the company nature, the regional economic level and company innovation strategies. This paper enriches the research related to the transformation of pharmaceutical enterprises and the change in the pharmaceutical industry.

## 2 Theoretical analysis and research hypothesis

### 2.1 Procurement policy and innovation input

Domestic and international studies have repeatedly been published on centralized drug procurement policies and corporate innovation. In terms of corporate operating profits, Mo and Zhou et al. postulated that the development of innovative drugs by Chinese companies would increase corporate profits by at least 50%, effectively compensating for the impact of lower profits from generic drugs after centralized Volume-Based procurement (ZHOU and TAN, 2021; Mo, 2022). Maniadakis & Pieter, from the perspective of the duality of action, argue that centralized drug procurement, on the one hand, significantly reduces drug prices and brings benefits to patients, but on the other hand, also leads to a profit game between generic and innovative drugs (PieterArnold and Steven, 2011; Maniadakis et al., 2018). Fei found through empirical research that the negative impact of centralized Volume-Based procurement policies on corporate profits began to emerge gradually. He suggested that generic drug companies invest more in R&D and have their new drug products as soon as possible to enhance their core competitiveness (Hua et al., 2022). Triulzi showed that recognizing the differentiation of drug values in tendering policies and implementing pricing policies that reward value-added drug development can encourage pharmaceutical manufacturers to innovate production processes and quality levels (Triulzi et al,. 2016). Hongfei observed that purchasing by the Chinese government organization led to marked price reductions in the labeled medicines, but the price distribution was too diffuse and the price ratios were not reasonable, requiring further and effective price regulation means (Long et al., 2022).

In terms of drug quality, In the gradual implementation of a drug-centralized purchasing policy in China, Chao became concerned about the quality of labeled generic drugs and had difficulty in ensuring clinical effectiveness and safety (Zhang et al., 2023). Hu argues that companies with high-quality generic drugs should receive a certain policy tilt in their quotations, In order to support high-quality generic drugs and original research and development drugs, the price of the drug can be reduced by more than 50%. The rate of reduction is calculated on the basis of the highest effective declared price of the corresponding specification in the Catalogue of Purchased Varieties. He considered it could eliminate worries and invest more resources in innovative research and developing new drugs (Hu, 2021). (China’s centralized drug procurement platform requires that if the price reported by a company is more than 1.8 times the lowest price, the company will be disqualified). Based on the single procurement of insulin, Zhang and Shao found that the selected pharmaceutical companies’ agreed procurement volume increased several times compared to their historical production. Inferring that the collective procurement brought development opportunities to some companies with high-quality generic drugs and would have more sufficient funds for innovative R&D in the future (Zhang et al., 2022). Wang and Jiao analyzed the motivation of enterprises to innovate and found that assessing whether to win a bid solely based on price would reduce the motivation of enterprises. If price, quality, and supply capacity were considered together, it could enhance enterprises’ confidence and promote further innovation by pharmaceutical enterprises (Wang et al., 2022a). Richard, based on the failed case of drug procurement in Kenya, it is concluded that a bidding process based primarily on price carries significant risks to the economy and society and is ultimately detrimental to the innovative development of companies and the reduction of distribution costs (Tren et al., 2009).

Regarding internal management, Li and Shen found that after participating in the centralized Volume-Based procurement bidding, Huahai Pharmaceuticals significantly reduced the number of sales staff, reduced sales costs, increased the number of R&D staff, and focused more on R&D innovation (Li and Shen, 2020). Yang and Li et al. suggest that encouraging technology mergers and acquisitions or mergers and acquisitions among pharmaceutical companies can better achieve the transition from generic to innovative pharmaceutical companies (Yang et al., 2019). Zhang et al. emphasize that the implementation of product differentiation strategies by pharmaceutical companies can alleviate the negative impact of uncontrollable profits of a single drug under the existing market competition system and increase the incentive for innovation (Zhang et al., 2017).

Regarding industrial concentration and supplier competition, Lu analyzed pilot vs non-pilot cities for centralized Volume-Based drug procurement with a double difference method, found that the medical institutions in the pilot cities met fine their commitments to agree on the amount of drug use, giving the providers the confidence to lower the price in exchange for their sales (Lu et al., 2022). Huang and Tao Huang and Tao combine the theory of industrial concentration with the practice of centralized national drug procurement. The concentration of talent and capital will improve the innovation ability of pharmaceutical companies (Huang and Tao, 2020). Gabriel and Rifat argued that procurement organizations need to consider the reputation of drug suppliers and drug quality and avoid relying exclusively on a single drug supplier, thereby reducing the incentive for that drug supplier to innovate (Seidman and Atun, 2017). Marcel suggests that sourcing from multiple competitively priced suppliers, rather than only from the supplier offering the lowest price, encourages more suppliers to remain in the market (Marcel et al., 2018). This approach helps to keep prices down over time, reducing the likelihood of stock-outs and allowing companies time and space to innovate.

Based on the above theoretical analysis, it can be concluded that the in-depth promotion of centralized volume-based purchasing of drugs in China has caused an increase in the intensity of innovation investment in pharmaceutical companies. By influencing their internal management, drug profits, production processes, technology, capital pooling, market share, competitors, drug quality, and many other aspects. Therefore, this paper proposes the hypothesis:

H1 The centralized volume-based procurement policy for drugs organized by the State promotes the selected pharmaceutical companies to increase the intensity of their investment in innovation.

### 2.2 Property rights, regional heterogeneity, and innovation input

The advancement of a policy across the country impacts different firms. Scholars such as Yi and Wang argue that enterprises have internal and external heterogeneity. Enterprise heterogeneity is the basis for a better understanding and appreciation of enterprise behavior (Yi et al., 2015). From the perspective of corporate property rights, China’s socialist market economy system determines a market pattern in which public ownership is the mainstay, and multiple ownership systems co-exist. The existence of different property rights of listed companies may lead to differences in corporate discourse, sensitivity, and responsiveness to policy formulation. From the perspective of the region where the enterprise belongs, China’s economic and social development is unbalanced and insufficient. There are significant differences in the economic level, the degree of marketization, and policy implementation power between regions. Based on this, this paper examines in depth the different effects of centralized Volume-Based procurement policy on innovation inputs from both property rights and regional perspectives.

Property rights are an inherent attribute of listed companies and significantly impact their business activities. According to Wang and Zhu, a significant proportion of managers of state-owned enterprises have political experience, and management will follow policies closely out of concern for future political performance (Wang et al., 2018). On the other hand, private enterprises are less subject to government intervention and are more focused on maximizing shareholders’ interests as their business goal. Chen Hong et al. argues that in terms of innovation capability, private enterprises can absorb or acquire advanced technologies from other countries through cooperation with research institutions, merging with target enterprises and sharing R&D costs, and further imitating and innovating on this basis. In contrast, state-owned enterprises may have more difficulty acquiring advanced technologies from developed countries due to changes in the political environment (Chen et al., 2022). According to Wen and Feng, state-owned enterprises have non-competitive managers due to diversified business objectives (Wen and Feng, 2012). In terms of non-economic factors of corporate governance, institutional investors have a limited voice and cannot effectively exert innovative effects. The author argues that the centralized Volume-Based procurement policy for state-organized medicines is a robust measure organized and implemented by the government to promote supply-side structural reform. The nature of state-owned enterprises determines that they should follow the direction of national development and pay more attention to the essential tasks of future high-quality development, stabilizing people’s livelihoods, and preserving employment. The measurement weight of economic factors will be considered less. In addition, from the perspective of political performance, it is a prominent political standpoint to meet the supply-side reform of the pharmaceutical industry and promote innovation in the industry by firming up the political direction in the industry change. On the other hand, privately listed companies will react with a certain lag to industry changes due to information asymmetry. In summary, this paper proposes the following hypothesis.

H2 The centralized volume-based procurement policy for drugs has promoted state-owned enterprises to increase investment in innovation more effectively than private enterprises.

The ability of pharmaceutical companies to innovate is closely linked to the state of the local economy. Generally speaking, the more economically developed a region is, the more institutional investors there are, the better the entrepreneurial and innovative atmosphere, and the more support it receives. For example, the high number of universities and research institutes belonging to the three major economic zones can provide more excellent local talents. The cities belonging to the three major economic zones are well built and provide convenient transportation routes and postal services. Drug procurement policies promote the upgrading of the pharmaceutical industry, which requires more financial and intellectual support for pharmaceutical companies. Therefore regional heterogeneity is worth studying. Cui and Chen found that the proportion of intangible assets to total assets in China’s Beijing-Tianjin-Hebei region, Yangtze River Delta region, and Pearl River Delta region is higher than the national average proportion, and the proportion of fixed assets to total assets is lower than the national average proportion (Cui and Chen, 2016). They concluded that the three economic regions attach more importance to intangible assets and have more advanced management concepts for intangible assets. Due to their high economic development, they have a higher investment cost for intangible assets, and the cost of investment in R&D innovation is higher. The efficiency and results of R&D are more pronounced. Hu and Li found that, through regional differences, R&D investment in the three major economic zones positively affects technical efficiency. As R&D investment increases year by year, the overall level of R&D technical efficiency in the three major economic zones increases yearly (Hu and Li, 2011). Guo found differences and trends in the agglomeration and regional agglomeration effects of the three economic zones based on the analysis of urban and regional agglomeration effects (Guo, 2010). In addition, based on the Statistical Analysis Report on the Operation of the Pharmaceutical Distribution Industry released by the Ministry of Commerce of the People’s Republic of China in previous years. The author calculated that the proportion of total pharmaceutical sales in the three major economic regions is approximately 50% each year, accounting for half of the total pharmaceutical sales in the country. For example, 44.8% in 2015, 45.3% in 2016, 43.1% in 2017, 49.9% in 2020 and 49.6% in 2021. It can be seen that the inclusion of administrative regions belonging to the three major economic zones in one group, and other administrative regions in another group has a certain scientific and reference value. In summary, this paper puts forward the following hypotheses (Beijing-Tianjin-Hebei: Beijing, Tianjin, and Hebei; Yangtze River Delta: Shanghai, Jiangsu, and Zhejiang; Pearl River Delta: Guangdong.)

H3 The centralized volume-based procurement policy for drugs has promoted pharmaceutical companies in the three major economic zones to increase their investment in innovation more effectively than those in the non-three major economic zones.

## 3 Methods

### 3.1 Sample selection

It was given that the State Council’s notice agreeing on the centralized Volume-Based drug procurement policy organized by the State and issued to each region was published on 1 January 2019. The first batch of selected results of centralized Volume-Based drug procurement in the alliance regions was also announced in September of that year. Based on the availability of data, the period 2015–2021 was selected for analysis in this paper, using the time of publication of the notification as the node. For the sample companies, the corresponding exclusions were made according to the following criteria:

ⅰ A sample of ST and *ST companies.

‘ST and *ST’refers to stocks of domestic listed companies that are subject to special treatment, also known as delisting risk warnings. Due to its possible delisting at any time, this paper does not do research on such companies.

ⅱ A sample of companies with missing data.

The reason for excluding the sample of companies with “missing data” is that empirical research requires operating data. If it is difficult to obtain the operating data of that listed company, then it is impossible to conduct the next step of the study, so it can only be excluded.

ⅲ A sample of “U” companies.

According to Article 2 of the Notice on Matters Relating to the Trading of Shares and Depositary Receipts on the Technology Venture Exchange issued by the Shanghai Stock Exchange: If an issuer is not yet profitable, its shares or depositary receipts will be marked with a “U”; this special mark will be removed if the issuer achieves profitability for the first time. This study is an empirical study that requires data on operating income, profit, and sales revenue of each company. Therefore, we can only exclude it.

### 3.2 Model design

China’s centralized drug procurement policies can lead to individual differences between selected and non-selected pharmaceutical firms and differences between pharmaceutical firms before and after the implementation of the policies. The above two types of differences provide room for this paper to conduct quasi-natural experiments using a double difference model to assess the impact of the policy on pharmaceutical firms’ innovation. Listed pharmaceutical companies selected in the first to sixth batches of drug procurement in Shanghai and Shenzhen A-shares are used as the experimental group. Other listed pharmaceutical companies not selected are used as the control group. Treat variables (Treat is a grouping variable with values of 0 and 1) distinguish between the experimental and control groups, with the experimental group taking a value of 1 and the control group taking a value of 0.

The year 2019 was taken as the first year of implementation of the drug procurement policy (In fact, 2018 was the first year of implementation, but considering that it was carried out at the end of the year, it was difficult to influence the market quickly, so this study positions 2019 as the first year.). The time variable was used to distinguish the time before and after the implementation of the drug procurement policy, with the year after the implementation of the policy taking the value of 1 and the year before the implementation of the policy taking the value of 0. Considering that there are significant differences in the innovation culture and strength of different pharmaceutical companies, and there are also apparent differences in the same pharmaceutical company in different years, this paper refers to Marianne and Zhou and others to construct a two-way fixed effects benchmark model (Marianne and Sendhil, 2003; Zhou and Chen, 2005).

Based on the aforementioned theoretical analysis and research hypothesis H1, in the empirical test, a double-difference benchmark model of the intensity of innovation input of enterprises by China’s centralized volume-based procurement policy for drugs was constructed as follows:

$${Input}_{i,t}=\alpha_{0}+\alpha_{1}{Policy}_{i,t}+\alpha_{2}X_{i,t}+\mu_{i}+\lambda_{t}+\varepsilon_{i,t}$$ (1)

where Input_i,t_ denotes the intensity of innovation investment by pharmaceutical company i in year t, measured as the ratio of R&D investment to operating revenue in the CSMAR database. The impact effect of China’s centralized drug procurement policy is measured by the interaction term Policy_i,t_ (Policy_i,t_ = treat_i,t_ × time_i,t_). If the estimated coefficient α_1_ is significantly positive, it indicates that there is a positive policy effect of the centralized Volume-Based drug procurement policy on the innovation input intensity of listed pharmaceutical companies; if the coefficient α_1_ is significantly negative, it indicates that there is a negative policy effect of the centralized Volume-Based drug procurement policy on the innovation input intensity of listed pharmaceutical companies; if the coefficient α_1_ is not significant, it indicates that the impact of the centralized Volume-Based drug procurement policy is insignificant or not effective at the moment.

According to the previous theoretical analysis, drug procurement policy has a positive effect on the innovation performance of listed pharmaceutical companies in China, and α_1_ should theoretically be significant and positive. X_i,t_ denotes a set of control variables affecting the innovation input intensity of pharmaceutical firm i in year t; μ_i_ and λ_t_ denote individual firm fixed effects and time fixed effects respectively; α_0_ and ε_i,t_ denote constant terms and random disturbance terms respectively. This paper uses STATA 17.0 software to analyze the data.

### 3.3 Variable selection and data sources

In this paper, the intensity of innovation investment (Input) is used as the explanatory variable, with a more considerable Input indicating a more significant proportion of R&D investment as a percentage of operating revenue of a pharmaceutical company. It indicates that the pharmaceutical company attaches greater importance to and invests more in innovation; Input data is obtained from the CSMAR Economic and Financial Research Database created by Shenzhen Sigma Data Technology Co.

In addition, the quality of innovation output (Newdrug) and quantity of innovation output (Patent) will be used as explanatory variables in subsequent studies. New drug and Patent data come from the Pharmaceutical Intelligence Network (PIN) built by Chongqing Kangzhou Big Data Co Ltd, one of the most authoritative platforms for extensive data services and empowerment in the health industry in China.

Meanwhile, control variables were set according to previous studies, which were shrhfd5, ibd, y1001b, size, tobinq, businessyear, sa, executives. control variables were measured in the same way in different double-difference designs. A top and bottom 1% winsorize treatment was applied to some variables. The specific variable names, variable codes, measurements, and data sources for this study are shown in Table 1.

**TABLE 1:** Variable measurements and sources.

Variable codes	Measurement	Sources
Input	R&D investment as a percentage of operating revenue	CSMAR
Newdrug	Number of clinical approvals for new drugs	PIN
Patent*	Number of patents granted for inventions	PIN
Sale-Revenue	The ratio of selling expenses to operating income	CSMAR
Profit*	Direct Information Disclosure	CSMAR
Policy	The product of treat and time	Calculations
Area	1 three economic regions; 2 non-three regions	ORGAN
Nature	1 State-owned; 2 Privately owned	CSMAR
Shrhfd5	Sum of the squared shareholdings of the top 5 largest shareholders of the company	CSMAR
Ibd	Independent directors as a percentage of directors	Calculations
Y1001b	1 = same person; 2 = different person	CSMAR
Size*	Direct Information Disclosure	CSMAR
Tobinq	Total market capitalisation/assets	CSMAR
Businessyear*	year of observation (current accounting period) - year of business establishment	CSMAR
Sa	SA = −0.737*Size+0.043*Size^2-0.040*Businessyear	CSMAR
executives*	Sum of the number of directors, supervisors and senior management	CSMAR

Note: If there are some missing data for the above variables, then this study will fill in those data by other means; the formula for calculating market capitalisation is: A shares*Current value of A shares at today’s closing price + B shares of domestically listed foreign shares*Current value of B shares at today’s closing price (Shanghai*CNY_USD, Shenzhen/HKD_CNY, converted to RMB) + (Total number of shares - RMB ordinary shares - B shares of domestically listed foreign shares)*(Ending value of total owners’ equity/Ending value of paid-in capital for the period) + Ending value of total liabilities for the period; * denotes taking logarithms. “Y1001b” stands for whether the general manager and the chairman are the same person.1 = same person; 2 = different person. “Sa” stands for degree of corporate finance constraints.

## 4 Empirical results

### 4.1 Descriptive statistics and analysis

Table 2 presents the results of descriptive statistics for the minimum, maximum, mean, and standard deviation of the selected variables and independent sample t-tests for the selected and non-selected pharmaceutical enterprises. It can be seen that the selected pharmaceutical enterprise sample shows better characteristics than the non-selected pharmaceutical enterprises in terms of innovation input intensity, quality of innovation output, and quantity of innovation output. In addition, the t-tests for the differences in means between the selected and non-selected pharmaceutical enterprises for all indicators, except Tobin’s Q, business year, Sale-Revenue, and corporate finance constraints, are significant, indicating that there are significant differences. Preliminary indications are that the research in this paper has some significance.

**TABLE 2:** Descriptive statistics and t-tests for selected variables.

Variables	Total sample (N = 987) NO. Min max mean S.D.					Selected (N = 224)	Non-selected (N = 763)	Average value
						Average value	Average value	Differences
Input	987	0.350	29.380	5.891	4.823	7.781	5.336	2.445***
New drug	987	0	90	1.104	4.665	3.071	0.527	2.545***
Patent	987	0	4.762	0.667	0.872	0.983	0.574	0.410***
Sale-Revenue	987	0.003	1.034	0.263	0.173	0.273	0.260	0.013
Profit	987	15.73	22.28	19.45	1.358	19.679	19.379	0.300***
Area	987	1	2	1.539	0.499	1.438	1.569	0.131***
Nature	987	1	2	1.745	0.436	1.688	1.761	0.074**
Policy	987	0	1	0.097	0.296	0.429	0	0.429***
Shrhfd5	987	0.016	0.507	0.139	0.100	0.120	0.145	−0.025***
Ibd	987	0.286	0.500	0.376	0.053	0.370	0.378	−0.008**
Y1001b	987	1	2	1.688	0.464	1.786	1.659	0.126***
Size	987	18.790	24.730	22.090	1.091	22.37	22.010	0.358***
Tobinq	987	0.938	10.500	2.695	1.760	2.632	2.714	−0.082
Businessyear	987	2.197	3.497	2.953	0.264	2.962	2.950	0.011
Sa	987	−4.428	−3.446	−3.910	0.203	−3.913	−3.910	−0.004
Executives	987	2.398	3.219	2.764	0.189	2.798	2.754	0.044***

Note: N is the number of samples.*p < 0.1, **p < 0.05, ***p < 0.01.

### 4.2 Baseline regression results for hypothesis H1

This section examines the impact of China’s centralized drug procurement policy on the share of R&D investment in operating revenue of selected listed pharmaceutical companies based on changes in the intensity of innovation investment. Table 3 reports the results of the baseline regressions for hypothesis H1, where columns (Burns, 2022), (Vitry, 2014), and (Kaur et al., 2021) are the results of the regressions controlling for individual fixed effects only, time fixed effects only, and individual and time fixed effects without adding any other control variables, respectively. The coefficients on the dummy variables for drug procurement policy are all significantly positive and reach the 1% level of significance concerning the intensity of investment in innovation. As mentioned in the previous literature, China’s centralized Volume-Based drug procurement policy pushes pharmaceutical companies to transform and upgrade, continuously increase their innovation efforts, and increase their investment in research and innovation. With a large influx of funds into biopharmaceutical or new drug R&D, companies will gain the initiative in future drug procurement and improve their market bargaining power, thus accepting the hypothetical H1.

**TABLE 3:** Baseline regression results for hypothesis H1.

	(1)	(2)	(3)	(4)	(5)	(6)
	input	input	input	input	input	input
policy	2.856***	3.512***	1.867***	1.841***	3.674***	1.775***
	(0.524)	(0.789)	(0.548)	(0.531)	(0.745)	(0.553)
shrhfd5				0.349	−1.364	0.984
				(3.068)	(1.446)	(3.141)
ibd				4.991	0.958	5.022
				(3.122)	(2.909)	(3.137)
y1001b				−0.579*	−1.010***	−0.557*
				(0.304)	(0.309)	(0.303)
size				−0.147	0.00750	−0.121
				(0.204)	(0.166)	(0.207)
tobinq				−0.0199	0.553***	−0.0605
				(0.106)	(0.0994)	(0.117)
businessyear				9.274***	−2.538	8.864**
				(2.500)	(2.258)	(3.569)
sa				5.037	1.064	6.131
				(3.125)	(2.929)	(3.731)
executives				1.125	0.634	1.139
				(1.186)	(0.998)	(1.191)
_cons	5.613***	5.549***	5.709***	−2.739	15.32**	2.123
	(0.0979)	(0.147)	(0.0995)	(7.746)	(6.644)	(16.94)
N	987	987	987	987	987	987
Id	Yes	No	Yes	Yes	No	Yes
Year	No	Yes	Yes	No	Yes	Yes
R2	0.716	0.065	0.728	0.732	0.142	0.733

Note: Robust standard errors in parentheses, *p < 0.1, **p < 0.05, ***p < 0.01.

Columns (Mo, 2022), (ZHOU and TAN, 2021), and (Maniadakis et al., 2018) are the regression results of controlling for individual fixed effects only, time-fixed effects, and controlling individual and time-fixed effects. In contrast, all other control variables are added respectively, and the procurement policy dummy variables all reach the 1% level of positive significance, verifying the robustness of the model (Burns, 2022).

In addition to the core explanatory variables, the estimation results of the control variables from column (Maniadakis et al., 2018) of Table 3 shows that the coefficient of the year of operation of the enterprise is significantly positive. There is a significant positive effect of the year of operation of the selected pharmaceutical enterprises on the intensity of their R&D investment, which implies that the longer the year of operation of the pharmaceutical enterprises, the stronger they are to cope with the policy changes and challenges. Secondly, the coefficient for the situation where the chairman and the general manager are concurrently appointed is significantly negative, indicating that the separation of the chairman and the general manager of the selected pharmaceutical companies can better maintain the effectiveness and independence of board supervision, enhance the role of the board of directors, focus on long-term interests and respond to policy changes on time.

### 4.3 Robustness tests

#### 4.3.1 Parallel trend test and dynamic effects analysis

Since implementing a centralized Volume-Based drug procurement policy is a continuous dynamic adjustment process, it is necessary to consider further the dynamic effect of centralized Volume-Based drug procurement on the innovation development of pharmaceutical enterprises in China. This section draws on the method of Beck et al. (Thorsten et al., 2010) to conduct parallel trend tests. Table 4 shows the results of parallel trend tests and regressions of the dynamic effects of the centralized Volume-Based drug procurement policy on the innovation input intensity of pharmaceutical enterprises.

**TABLE 4:** A parallel trend test of the impact of centralized drug procurement policy on the intensity of innovation investment of listed pharmaceutical companies.

	(1)
	input
coeff2016	0.141
	(1.046)
coeff2017	0.666
	(0.870)
coeff2018	1.138
	(0.921)
coeff2019	1.573
	(0.961)
coeff2020	2.462**
	(1.083)
coeff2021	2.764***
	(1.057)
N	987
Control variables	Yes
Year	Yes
Id	Yes
R2	0.735

Note: Robust standard errors in parentheses, *p < 0.1, **p < 0.05, ***p < 0.01.

A particularly important assumption for the use of double difference models (DID) is that “parallel trends” are satisfied. Whether using graphs, regressions, or descriptive statistics, it needs to be shown that the experimental and control groups must be comparable before a shock or policy occurs. This is because the performance of the control group is assumed to be the counterfactual of the experimental group. It can be visually reflected that the coefficients of the double interaction terms (coeff 2016, coeff2017, coeff2018) before the implementation of the pooling policy in 2019 are not significant, indicating that the trend of change in the treatment and control groups before the implementation of the pooling policy is the same and there is no significant difference. The coefficient of the double interaction term in 2019 after implementation is marginally insignificant. The coefficients of the double interaction terms in subsequent years (coeff2020, coeff2021) are significantly different from zero, indicating that the selected pharmaceutical enterprises in the treatment group have a greater intensity of innovation input than the non-selected pharmaceutical enterprises in the control group. Therefore, the DID model used in this study satisfies the parallel trend assumption and the effect of policy implementation has some persistence.

**FIGURE 1:**
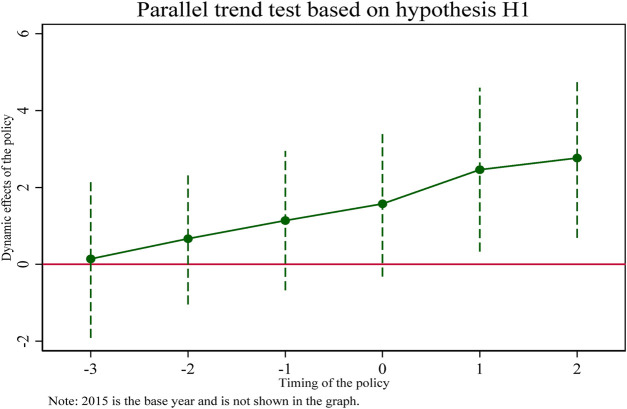
Dynamic effects.

In addition, this section reflects the regression results in column (Burns, 2022) of Table 5 in Figure 1 and plots the 95% confidence intervals of the regression coefficients for each year, from which it can be seen that until 2019, the estimated coefficients of the double interaction term fluctuate up and down around the value of 0 and are insignificant. While 3 years after the implementation of the pooling policy, the line of marginal effects rapidly slopes to the upper right, with increasing positive dynamic effects, indicating that the collective harvesting policy has caused a significant positive shock impact on the innovation input intensity of the selected pharmaceutical enterprises. This further confirms the paper’s hypothesis H1, that the pooling policy promotes selected pharmaceutical enterprises to increase their R&D investment intensity.

**TABLE 5:** A PSM-DID test of the impact of centralized drug procurement policy on the intensity of innovation investment of listed pharmaceutical companies.

	(1)
	input
policy	2.012***
	(0.659)
_cons	2.333
	(34.67)
N	462
Control variables	Yes
Year	Yes
Id	Yes
R2	0.771

Note: Robust standard errors in parentheses, *p < 0.1, **p < 0.05, ***p < 0.01.

#### 4.3.2 PSM-DID test

**FIGURE 2:**
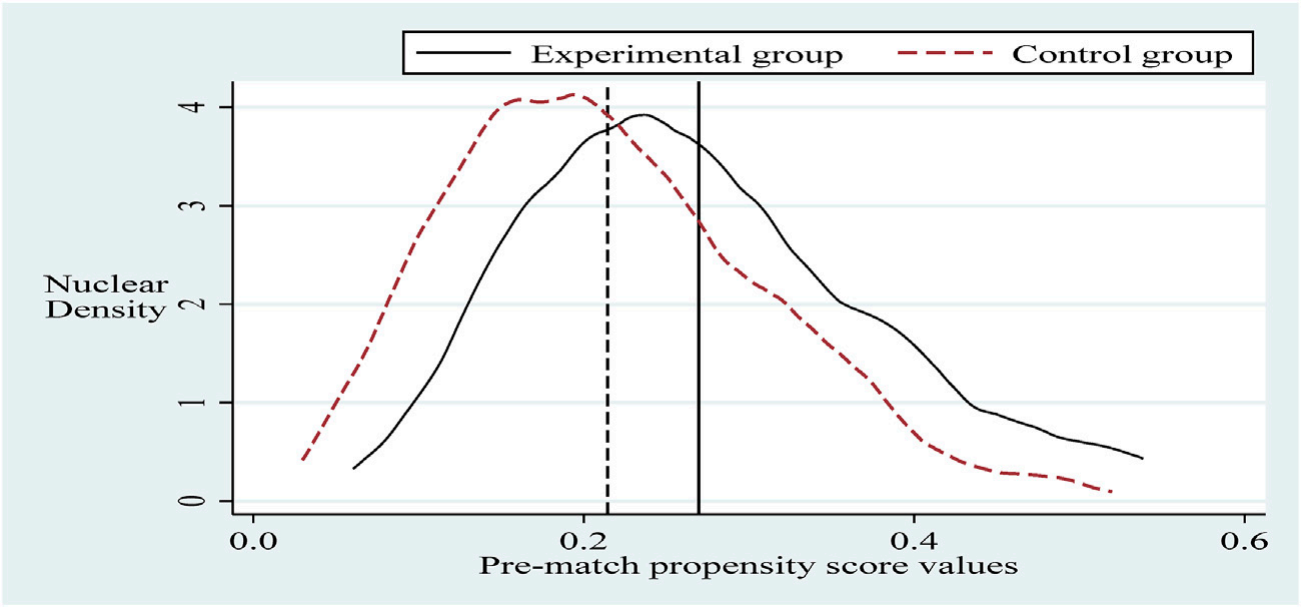
Pre-match propensity score values.

**FIGURE 3:**
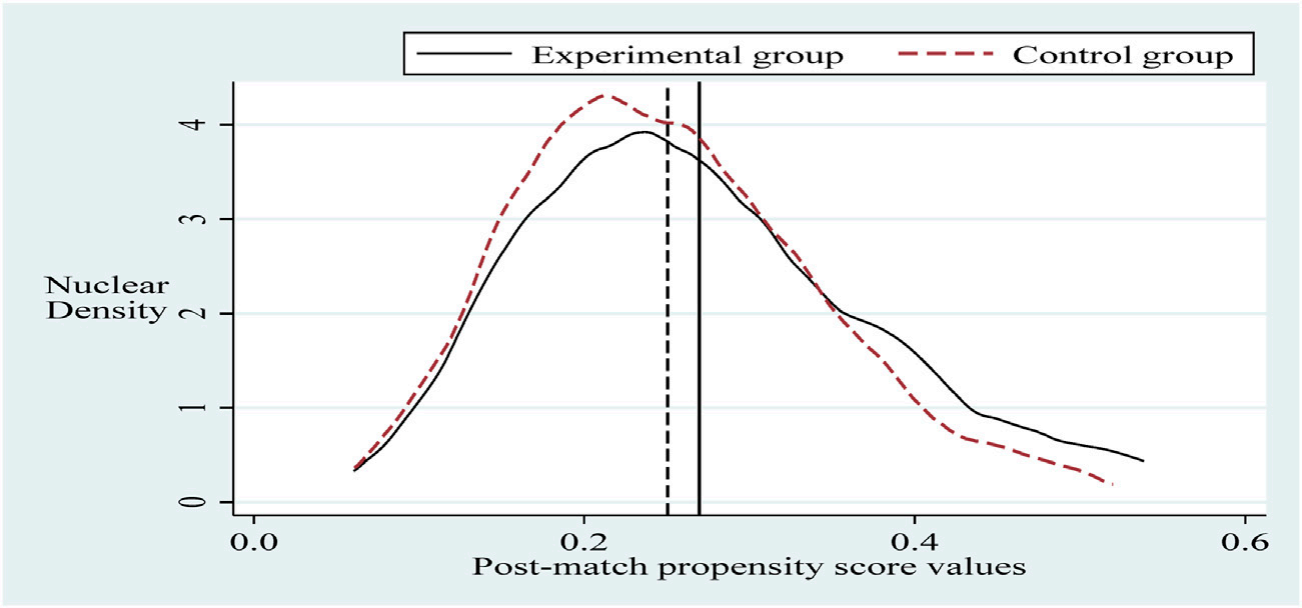
Post-match propensity score values.

To alleviate the problem of sample selection bias, this section refers to the practice of Sun and Ge. Further, it applies the propensity score matching-dual difference (PSM-DID) method (PSM nearest neighbor matching is the process of finding one or more individuals in the control group for each individual in the experimental group to match with.) with a 1:2 nearest neighbor matching method without put-back for robustness testing (Based on the sample size of the experimental group and the sample size of the control group, it is more reasonable to choose 1:2 in this paper. The reason is that if we choose 1:1, the final matching sample is smaller and the estimated variance is larger. If we choose 1:2 or 1:3 or others, the similarity between the third and fourth control group individuals matched with the experimental group individuals decreases, and thus the estimation bias increases.). Selecting the control variable in the same innovation input intensity benchmark regression as the matching variable and setting the caliper to 0.01, applying the Logit method was used for probability estimation. Propensity scores were obtained before and after matching (Sun and Ge, 2021). It can be seen that there is a specific selection bias between the sample treatment group and the control group before matching, and after matching, the sample treatment group is the same as the control group, as shown in Figures 2, 3. Next, hypothesis H1 was re-estimated for the sample after eliminating the selection bias, and the results are shown in column (Burns, 2022) of Table 5. The policy dummy variable reaches the 1% level of positive significance. The results of the robustness test are consistent with the previous section.

#### 4.3.3 Placebo test

**FIGURE 4:**
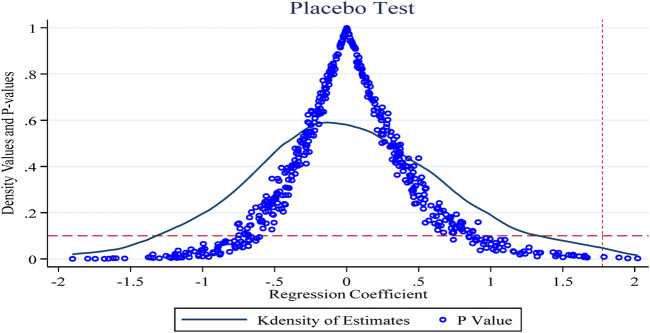
Placebo test.

To exclude the effects of endogeneity of policy shocks and individual firm heterogeneity on the study findings, this section conducts a placebo test repeated 500 times concerning Ningbo et al. (Ning et al., 2020). Figure 4 shows the results of the placebo test with the innovation R&D input intensity indicator as the explanatory variable. The horizontal and vertical coordinates of the dots in the figure indicate the coefficients and *p*-values of the policy dummy variables in the random combination case, and the curves show the kernel density distribution of the estimated coefficients, with the horizontal dashed line being the significance level of 0.1 and the vertical dashed line being the true estimate of the double-difference model of 1.775 (the baseline regression Table 3 column 6 of the policy coefficient estimates). As shown, the *p*-values for the random sample are generally above 0.1 (insignificant at the 10% level) and the coefficient estimates obtained based on the random sample are also generally smaller than those obtained in the benchmark regression, which further suggests that the results obtained in this paper are robust.

### 4.4 Heterogeneity tests

The previous regression results suggest that the centralized drug procurement policy significantly increases the intensity of innovation investment by the selected pharmaceutical companies. However, it is still unknown how the centralized Volume-Based drug procurement policy affects innovation in pharmaceutical firms in the context of regional and firm property rights. The following section will further explore the mechanism of action within the ‘black box.'

#### 4.4.1 Regional distribution

The above analysis has verified the impact of the harvesting policy on selected listed pharmaceutical companies at an industry-wide level. However, as China is a developing country with uneven regional development, the effect of policy implementation is often heterogeneous at the regional level. For many years, the Ministry of Commerce of China has published the “Statistical Analysis Report on the Operation of the Pharmaceutical Distribution Industry,” which contains statistics on the regional distribution of sales from multiple perspectives. We have found that the annual sales of the three major economic zones account for around 40%–50% of total national sales, which is relatively stable and has particular research significance. In terms of spatial distribution, the three economic zones are are located in northern China, southern China and eastern China, which can drive the development of each region and of course attract the investment of resources from each region., with developed transportation, high research, and management level, an excellent economic base, and many enterprises.

Other regions have a weaker economic base and fewer enterprises than the three economic zones, but they are rich in resources and have great potential for development. In this paper, we further investigate the effects of the state organization’s centralized Volume-Based procurement policy on the innovation investment of regional listed pharmaceutical companies through the double-difference and mediating effect models. As in columns (Burns, 2022) to (Vitry, 2014) of Table 6, the double interaction term is positively significant at the 5% level for the three major economic regions and insignificant for the non-three major economic regions. It indicates that the centralized Volume-Based drug procurement policy has a better effect on promoting the intensity of innovation investment of pharmaceutical enterprises in the three major economic zones than in the non-three major economic zones.

**TABLE 6:** The impact of heterogeneity on the intensity of innovation investment in listed pharmaceutical companies.

	(1)	(2)	(3)	(4)
	input	input	input	input
Three Economic regions	1.759**			
	(0.701)			
Non-three economic regions		1.321		
		(0.824)		
State-owned and state-controlled			1.759**	
			(0.701)	
Private enterprise				1.321
				(0.824)
_cons	4.523	5.432	4.523	5.432
	(16.96)	(16.99)	(16.96)	(16.99)
N	987	987	987	987
Control variables	Yes	Yes	Yes	Yes
Id	Yes	Yes	Yes	Yes
Year	Yes	Yes	Yes	Yes
R2	0.731	0.729	0.731	0.729

Note: Robust standard errors in parentheses, *p < 0.1, **p < 0.05, ***p < 0.01.

#### 4.4.2 Nature of enterprises

For the pharmaceutical industry, introducing national policies has a significant impact on enterprises, such as the promotion of GMP and GSP, medical insurance, and the primary drug catalog. This centralized Volume-Based drug procurement is no exception. Although private pharmaceutical companies can voluntarily make strategic adjustments according to their own development goals, combined with the current situation of the company, and freely control the pace of development, it is easy to seize market opportunities in the market.

However, in terms of keeping up with national policy acceptance, safeguarding people’s livelihood, and guiding changes in the industry, state-owned pharmaceutical enterprises are more advanced. Secondly, state-owned enterprises have easy access to information on the future direction of policies and the strength of their promotion. Columns (Kaur et al., 2021) and (Mo, 2022) of Table 6 show the results of the regressions grouped according to the nature of the enterprises. The regression results show that the double interaction term for state-owned and state-controlled is positively significant at the 5% level. At the same time, the private sector is insignificant, and the centralized procurement policy to promote innovation investment intensity is better at the level of state-owned pharmaceutical enterprises.

### 4.5 Mechanism test

The previous section found that the centralized drug procurement policy significantly promoted the selected pharmaceutical companies to increase their investment in innovation. The increase in innovation investment by the selected pharmaceutical companies may be due to the increase in profits from the increased market share of the selected products. Alternatively, the lack of profits after winning the pooled procurement may encourage companies to increase their R&D efforts in biologics and innovative drugs. Also, it may be due to the increase in innovation investment by reducing the relative sales costs. The views of scholars and experts differ on the above points, but they remain in qualitative research and less in empirical evidence. The impact of internal corporate mechanisms is not yet known, so the sample chosen for this paper is of some relevance. From the perspective of the sales expense ratio, enterprises save money by reducing the sales expense ratio to encourage corporate innovation, increase investment and improve the level of corporate innovation and innovation capacity. In addition, from the perspective of business profits, high profits promote enterprises to have more strength to carry out innovation work, improve the tolerance of innovation risk, and thus obtain high-quality innovation results. Alternatively, centralized Volume-Based drug procurement policy have not yet divided the “pie,” resulting in companies, as one of the main stakeholders, interrupting the national distribution of mature drug sales networks, reducing profits. So, it opted to enter biologics and innovative drugs to increase its bargaining power in the market.

To test the above two mechanisms, this paper uses the stepwise regression method with the coefficient product Sobel test to test the mediating effect. The specific model is as follows.

$$Sale/Revenue/{profit}_{i,t}=\delta_{0}+\delta_{1}{Policy}_{it}+\delta_{2}X_{i,t}+\mu_{i}+\lambda_{t}+\varepsilon_{i,t}$$ (2)

$${input}_{i,t}=\theta_{0}+\theta_{1}{Policy}_{it}+\theta_{2}Sale/Revenue/{profit}_{i,t}+\theta 3X_{i,t}+\mu_{i}+\lambda_{t}+\varepsilon_{i,t}$$ (3)

The regression results of model (2) and model (3) are presented in Table 7. The coefficients of δ_1_ are significant at the 10% level when the explanatory variable in the model (3) is sale/revenue, indicating that the selected firms save money and encourage innovation by reducing the sales cost rate. From column (2), the coefficient of policy is more significant after controlling for the mediating variable sale/revenue. However, the coefficient of the mediating variable sale/revenue, is not significant. To explore the reason, this paper uses the Sobel test for mediating effect and finds that the *p*-value is less than 0.01, indicating that the mediating effect holds, and the calculated mediating effect accounts for 8.98% of the total effect, see Table 8. From column (3) and column (4), it can be seen that when the explanatory variable in model (The coefficient of δ_1_ is significant at the 5% level when the explanatory variable in model (Kaur et al., 2021) is profit; when controlling for profit as the mediating variable, the coefficient of policy is positively significant at the 1% level and the coefficient of profit is negatively significant at the 1% level, which is sufficient to show that China’s current centralized Volume-Based drug procurement policy has not yet co-ordinated the interests of all stakeholders, and enterprises’ profits are damaged and enterprises There is a certain passivity to change the business philosophy and increase innovation in order to obtain better bargaining power and growth.

**TABLE 7:** Mechanism tests.

	(1)	(2)	(3)	(4)
	sale/revenue	input	profit	input
policy	0.0217*	1.715***	0.299**	2.052***
	(0.0120)	(0.543)	(0.123)	(0.538)
sale/revenue		2.763		
		(2.076)		
profit				−0.908***
				(0.187)
_cons	−1.629***	6.623	7.970	4.831
	(0.470)	(17.45)	(5.123)	(15.75)
N	987	987	906	906
Control variables	Yes	Yes	Yes	Yes
Id	Yes	Yes	Yes	Yes
Year	Yes	Yes	Yes	Yes
R2	0.824	0.735	0.805	0.763

Note: Robust standard errors in parentheses, *p < 0.1, **p < 0.05, ***p < 0.01.

**TABLE 8:** Sobel test.

Sobel test (cost of goods sold ratio)				
Coef	Std	Err	Z	P>|Z|
Sobel	0.392	0.136	2.874	0.00406
Goodman-1	0.392	0.137	2.855	0.00430
Goodman-2	0.392	0.135	2.892	0.00382
The proportion of total effect that is mediated				0.0898
Ratio of indirect to direct effect				0.0987
Ratio of total to direct effect				1.099

## 5 Further examination: how centralized drug procurement policies affect the innovation output of listed pharmaceutical companies

### 5.1 Drug procurement policy and innovation output

As mentioned earlier, centralized Volume-Based drug procurement policy have significantly increased the intensity of firms’ innovation inputs. It is worth considering how the increase in innovation inputs has changed firms’ innovation outputs. It is not enough to consider only the relationship between inputs and outputs without considering corporate strategies in the context of centralized Volume-Based drug procurement. In the context of implementing the centralized Volume-Based procurement policy for drugs organized by the state, many large pharmaceutical companies whose ace products did not win the tender have put all their efforts into researching and developing innovative drugs. Their former negative innovation strategy has become more innovation-driven. Some companies are committed to an indirect innovation strategy through inter-company technology mergers or acquisitions to achieve product differentiation and diversification. Some Enterprises have pursued technological innovation strategies, focusing their vision on low price, high quality, and sufficient output of winning drugs, and vigorously developing new technologies and techniques for generic drugs. Therefore, the centralized Volume-Based drug procurement policy has dramatically influenced the choice of innovation strategies of enterprises based on their strength and the surrounding environment.

The linkages and performance of firms’ innovation strategies and innovation outputs under the influence of centralized Volume-Based drug procurement policy have also attracted numerous scholars to explore. According to Chen, the development strategies of pharmaceutical firms can be classified as autonomous, integrated, and joint innovation, and the quality of their innovation output often differs (Chen, 1991). Marisa found that Portuguese firms lacked an open innovation strategy, making their innovation outcomes lack international competitiveness and sustainable competitive advantage (Marisa and Sílvia, 2019). He found through a questionnaire that clear innovation strategies help to improve innovation performance, and false innovation strategies even weaken the positive impact of other suitable mechanisms on innovation performance (He et al., 2016). Hu&Jefferson, when analyzing innovation policies, patent system protection, and promotion, Hu&Jefferson found that firms choose positive development strategies, increase their innovation R&D investment and promote the quality of their patents (Albert et al., 2008). conti& Haeussler showed that if a firm has a market entry strategy, it uses the number of patents as a market signal to its target customers to prove its competitive strength but ignores the quality of patents and the industrialization of patents (Annamaria et al., 2013; Carolin et al., 2014). According to Xu and others, the dynamic adaptation of firms to their environment inevitably creates a link between outcomes, evolution, and strategy, i.e., the innovation output of firms is inextricably linked to the choice of innovation strategy (Xu et al., 2014). Therefore, the difference in the innovation output of pharmaceutical companies is a concentrated expression of the strategy of pharmaceutical companies, and different strategic choices will make companies’ innovation output vary greatly.

Then, Chinese-listed pharmaceutical companies participate in centralized drug procurement in the face of the integration and optimization of the national market by the centralized Volume-Based drug procurement policy. Will they choose to lay out high-end generic drugs and strictly control the quality and safety of their products concerning the consistency evaluation standards of generic drugs? Or will they accelerate the development of innovative drugs to improve national and international competitiveness? Or will they take improved new drugs as the main direction of attack, with equal emphasis on risks and expected returns? Alternatively, will they provide stable generics as the main focus, with an innovative expansion path, or seek corporate mergers, technology mergers, and project integration to improve innovation performance in a short period, all of which is unknown? There is a paucity of research involving drug procurement policies’ impact on pharmaceutical companies’ output performance and a lack of empirical studies. Therefore, the following hypotheses are proposed in this paper from an empirical perspective.

H4 China’s centralized volume-based procurement policy promotes a change in the innovation strategy of selected pharmaceutical companies, emphasizing innovation quality over quantity.

H5 China’s centralized volume-based procurement policy promotes a change in the innovation strategy of selected pharmaceutical companies, emphasizing innovation quantity over innovation quality.

H6 China’s centralized volume-based procurement policy promotes a change in the innovation strategy of selected pharmaceutical companies, placing equal emphasis on innovation quality and quantity.

### 5.2 Multiple regression based on innovation output hypothesis

The aforementioned theoretical analysis shows that as drug procurement policies are advanced, there will be significant differences in the innovation output of companies. The pharmaceutical industry is unique in that it attaches importance to protecting intellectual property rights and timely information, such as clinical filings, as required by law. We can differentiate and quantify the quality differences in the innovation output of pharmaceutical companies based on specific criteria to obtain the proper level of innovation and thus help address the question of how the national centralized drug procurement policy affects innovation output. However, the question of choosing appropriate output indicators to measure the quality of innovation output after centralized Volume-Based drug procurement is an urgent issue to be addressed.

Yang and Wu took the number of invention patent applications by pharmaceutical companies as innovation output from the input-output activities of enterprises (YANG, 2020). Wang and Zhu used the number of patents applied for and eventually granted by enterprises in the same year to measure the quantity of enterprise innovation. The number of other citations of individual patents applied for and eventually granted in the same year is a measure of enterprise innovation quality, which is somewhat innovative (Wang et al., 2022b). Tang and Zhou selected the number of R&D personnel and R&D expenditure as input indicators and the number of new patents and business income as output indicators (Tang et al., 2022). Du and Zhang argue that the number of patent applications needs to be verified and audited in detail, so the number of patent applications does not represent innovation output. The percentage of new product sales does not consider the knowledge achievements at the theoretical level; it is more reasonable to choose the logarithm of technology contract turnover as an indicator to measure regional innovation output (Du and Zhang, 2022). According to Yin and Chen, innovation output can be decomposed into the innovation generation and innovation transformation stages for the technology-driven pharmaceutical industry, measured by the number of annual patent applications and primary business income, respectively (Yin and Chen, 2016). Comparing the data of the last 3 years, Chen can find that the number of new drug clinical trials has increased year by year after the implementation of the collection policy: in 2019, 2020, and 2021, the proportion of new drug clinical trials is 52.7%, 56.6%, and 60.5% respectively. In 2021, for example, 3,358 clinical trials will be registered on China’s drug clinical trial registration and information disclosure platform, and 2,033 new drug clinical trials will be registered. New drug research and development is the highest productivity and innovation ability of pharmaceutical enterprises, and the increase in new drug clinical applications is of great significance.

This paper shows the number of patents granted as an output indicator of innovation quantity. The number of new drug clinical applications as an output indicator of innovation quality. They are chosen to investigate whether the centralized Volume-Based procurement policy for drugs organized by the State has a significant impact on the innovation output of pharmaceutical enterprises, and a model is constructed as follows.

$${Newdrug}_{i,t}=\beta_{0}+\beta_{1}{Policy}_{it}+\beta 2X_{i,t}+\mu_{i}+\lambda_{t}+\varepsilon_{i,t}$$ (4)

$${Patent}_{i,t}=\gamma_{0}+\gamma_{1}\;{Policy}_{it}+\gamma 2X_{i,t}+\mu_{i}+\lambda_{t}+\varepsilon_{i,t}$$ (5)

where Newdrug_i,t_ denotes the quality of innovation output of pharmaceutical firm i in year t, measured by the number of new drug clinical applications filed by pharmaceutical firms. patent_i,t_ denotes the quantity of innovation output of pharmaceutical firm i in year t, measured by the number of invention patents granted to pharmaceutical firms. Models (Mo, 2022) and (ZHOU and TAN, 2021) choose the same set of control variables and fixed effects as in model (Burns, 2022). β_0_, γ_0_ denote constant terms and ε_i,t_ are random disturbance terms.

This section examines the impact of China’s centralized drug procurement policy on the innovation philosophy of selected listed pharmaceutical companies based on a dual perspective of quality and quantity of innovation output. Table 9 reports the results of the benchmark regressions, where columns (Burns, 2022) and (Kaur et al., 2021) are the results of regressions controlling for individual and time-fixed effects only without adding other control variables, respectively. Collective procurement policies are closely related to firms’ innovative ideas, with the coefficient of the dummy variable for the centralized Volume-Based drug procurement policy for state-organized medicines being significantly positive at the 5% level of significance for the quality of innovative output, and the coefficient of the dummy variable for the centralized Volume-Based drug procurement policy for state-organized medicines being significantly negative at the 1% level of significance for the quantity of innovative output. Through the previous theoretical analysis and result verification, we can find that the innovation strategy of the selected pharmaceutical enterprises has changed significantly after the centralized Volume-Based drug procurement, and their innovation output has certain characteristics of suppressing the quantity of innovation and focusing on the quality of innovation. Thus hypotheses H5 and H6 are rejected, and hypothesis H4 is accepted, i.e., the centralized Volume-Based drug procurement policy of the state organization promotes the selected pharmaceutical enterprises to change their innovation strategy and focus on the quality of innovation rather than the quantity.

**TABLE 9:** Regression results for the innovation output hypothesis.

	(1)	(2)	(3)	(4)
	New drug	New drug	patent	patent
Policy	1.813**	1.658**	−0.400***	−0.392***
	(0.902)	(0.831)	(0.0987)	(0.0996)
_cons	0.928***	49.61*	0.705***	−2.562
	(0.126)	(26.73)	(0.0202)	(3.360)
N	987	987	987	987
Control variables	NO	Yes	NO	Yes
Year	Yes	Yes	Yes	Yes
Id	Yes	Yes	Yes	Yes
R2	0.535	0.546	0.627	0.629
adj. R2	0.453	0.461	0.562	0.560

Note: Robust standard errors in parentheses, *p < 0.1, **p < 0.05, ***p < 0.01.

Columns (Vitry, 2014) and (Mo, 2022) further demonstrate the robustness of the model by showing that the centralized Volume-Based drug procurement policy dummy variable remains significantly positive in terms of quality of innovation output and negative in terms of quantity of innovation output after the inclusion of control variables. The above regression results validate hypothesis H4 that the centralized Volume-Based drug procurement policy of the state organization significantly increases the importance of the quality of innovation and reduces the demand for quantity of innovation among the selected pharmaceutical companies.

## 6 Conclusion and recommendations

This paper applies two-way fixed effects double difference estimation to a quasi-natural experiment using the national drug centralized Volume-Based procurement policy. Empirically investigates the impact of the centralized Volume-Based drug procurement policy on the innovation of listed companies in China’s pharmaceutical industry and obtains the following conclusions:

Firstly, the centralized Volume-Based drug procurement policy has promoted a significant supply-side structural reform in China’s pharmaceutical industry, making the previous situation of relying solely on low-end generic drugs to “win the world” disappear. The proportion of R&D expenditure to operating revenue has been increasing. In the era of centralized Volume-Based drug procurement, poor-quality generic drugs are eliminated, generic drugs of good quality obtain general manufacturing profits, and innovative drugs can obtain higher pricing power. Although corporate innovation, especially the development of new drugs, is risky, the author holds a positive attitude towards the innovation of Chinese pharmaceutical companies in the coming years.

Secondly, the centralized Volume-Based drug procurement policy has a heterogeneous impact on listed companies of different business natures in China. It showed that listed companies of state-owned nature are more likely to obtain the right to information and are also more able to keep up with policy changes and show higher motivation. From the empirical results, the R&D investment intensity of state-owned listed companies is better than that of private companies. Private companies may have a lack of understanding of the policy in the early stage of the promotion of the collection policy, which leads them to lose a certain degree of motivation. However, with the deepening and normalization of the collection, relying on the convenience of privately listed companies in financing, merging, and other financial behaviors, they can quickly enhance their innovative R&D capabilities and improve the industry’s competitiveness.

Thirdly, centralized Volume-Based drug procurement policy has a heterogeneous regional effect on the innovation of listed pharmaceutical companies. The effect of the intensity of innovation investment in China’s three economic regions is better than that of the non-economic regions. The reasons for this are a large number of pharmaceutical companies, the large size of the companies, the high market share of the enterprises, the substantial capital, the large reserve of scientific researchers, a large number of scientific research institutions and research colleges, the high level of regional GDP, and the excellent innovation atmosphere belonging to the three economic zones.

As innovation continues, innovation exchanges across the pharmaceutical industry will become more frequent, ultimately promoting the innovative progress of pharmaceutical companies nationwide.

Fourthly, through mechanism testing, the author found that the selected enterprises save money by lowering their own sales expense ratio as a mediating way to increase innovation investment. This mediating effect accounts for 8.98% of the total effect. In addition, when controlling for operating profit as the mediating variable and R&D investment as the outcome variable, the coefficient of centralized Volume-Based drug procurement policy is positively significant. Operating profit is negatively significant, indicating that the promotion of centralized Volume-Based drug procurement breaks the original interest pattern and temporarily reduces the operating profit of enterprises. It also encourages enterprises to rely on their original capital or financing and increase innovation investment to improve profits.

Fifth, the centralized Volume-Based drug procurement policy has influenced enterprises’ innovation output and strategic choices. With the in-depth promotion of the pooling policy, more and more companies understand that “innovation is the lifeline of companies.” The innovation strategies of selected pharmaceutical companies have undergone profound changes. There is a large gap between the gold content of ordinary patents and new drug clinical applications for pharmaceutical companies. The empirical results show that the centralized Volume-Based drug procurement policy has changed companies’ innovation output in recent years, with R&D characteristics that suppress the quantity of innovation and increase its quality.

Based on the above conclusions, the recommendations of this paper are as follows:

Based on the findings of the study, it is recommended that companies take a proactive approach to innovation. The profits of low-tech products such as generics will be further squeezed, and only with an efficient R&D innovation team and innovative drugs will they be able to gain a foothold in the future Chinese pharmaceutical market. The rich financial and human resources of the three major economic zones can provide help for the development of enterprises. Companies should strengthen their internal institutions and resource integration capabilities, save money by reducing the sales expense ratio and other means, and expand R&D investment.

Based on the findings of the study, it is recommended that the government should introduce enterprise R&D subsidy policies to reduce the risk of enterprise innovation and R&D, and also help enterprises adapt to the changes in the industry due to the centralized volume-based drug procurement policy. In addition, regional differences among pharmaceutical companies should be reduced, cross-regional cooperation among pharmaceutical companies should be encouraged and promoted, and technology and talent exchange should be enhanced. The government should pay more attention to private companies than state-owned and state-controlled enterprises, and help solve the difficulties they encounter when developing innovations. Of course, it is essential to improve the procurement process and strengthen the process supervision.

In addition, for countries like China that rely on procurement platforms, this paper recommends promoting the standardization of procurement platforms and improving the procurement system. Prevent the risk of corporate default and ensure the quality and quantity of drug and medical device supply.

This study has some limitations and several deficiencies. Firstly, this study takes all listed companies in the pharmaceutical manufacturing industry as the sample (except ST and *ST companies, companies with missing data, and “U" companies) according to the industry classification of the China Securities Regulatory Commission. Considering China’s centralized Volume-Based Drug Procurement, listed companies engaged in medical services or medical devices are under much less pressure to innovate than those engaged in drug R&D and manufacturing. The study did not distinguish between business scope. Second, the subject of this study is actually the affected listed pharmaceutical companies, and the description of the China’s centralized Volume-Based Drug Procurement itself is relatively few. Finally, Volume-Based drug procurement has been conducted several times. It is difficult to quantify whether there is an impact on the incentive to innovate when a listed company is selected the first time and then loses in the next procurement.

## Data Availability

The original contributions presented in the study are included in the article/supplementary materials, further inquiries can be directed to the corresponding author.
